# L’érythrocytose après transplantation rénale: étude rétrospective à propos de 11 receveurs

**DOI:** 10.4314/pamj.v5i1.56189

**Published:** 2010-04-29

**Authors:** Zbiti Najoua, Rhou Hakima, Benamar Loubna, Ezaitouni Fatima, Bayahia Rabia, Ouzeddoun Naima

**Affiliations:** 1 Service de Néphrologie Dialyse Transplantation rénale CHU Ibn Sina, Rabat, MAROC

**Keywords:** érythrocytose, transplantation rénale, traitement, pronostic

## Abstract

**Introduction:**

L’érythrocytose après transplantation rénale (ETR) survient chez 5 à 20 % des transplantés rénaux. Le but de notre travail est de déterminer la prévalence de l’érythrocytose chez les transplantés rénaux et de connaître les facteurs de risque d’apparition de cette érythrocytose et son impact sur la fonction rénale.

**Méthodes:**

Notre étude est rétrospective portant sur 74 transplantés rénaux. Nous avons distingué 2 groupes de patients selon la présence ou non de l’érythrocytose, celle-ci étant définie par un taux d’hématocrite supérieur à 51% selon les recommandations de Kidney Disease Improving Global Outcomes (KDIGO).

**Résultats:**

L’érythrocytose est retrouvée chez 11 patients, soit une prévalence estimée à 14,8%. On note une nette prédominance masculine (63,6% vs 36,4%). L’âge moyen de nos patients était de 40 ± 11 ans pour le Groupe1 vs 36 ± 13 ans pour le Groupe 2. La durée de transplantation rénale était de 42 ± 33 mois pour le groupe1 vs 36±26 mois pour le groupe2. Le délai d’apparition de l’érythrocytose par rapport à la transplantation rénale est de 9±7 mois. Aucun facteur de risque n’a été retrouvé dans notre série. Quatre patients ont nécessité des saignées et 9 ont été traités par un inhibiteur de l’enzyme de conversion. La rémission a été notée chez tous les patients. On n’a pas noté de complication thrombo-embolique.

**Conclusion:**

L’érythrocytose après transplantation rénale reste une complication, le plus souvent, bénigne. Le traitement est basé sur les saignées mais surtout les inhibiteurs de l’enzyme de conversion.

## Introduction

L’érythrocytose après transplantation rénale (ETR) est une complication qui survient dans 5 à 20% des cas. Son étiopathogénie reste mal connue mais il s’agit probablement d’une hyperproduction d’érythropoïétine par le greffon et par les reins natifs.

L’objectif de notre étude est d’évaluer la prévalence de l’ETR afin de déterminer les facteurs de risque de son apparition et de connaître son impact sur la fonction rénale.

## Méthodes

Il s’agit d’une étude rétrospective portant sur 74 transplantés rénaux (Tr) suivis au service de néphrologie du CHU Ibn Sina de Rabat, de Juin 1998 à Août 2008. Nous avons distingué 2 groupes de patients selon la présence ou non de la PG: (a) Groupe 1: patients ayant développé une érythrocytose, (b) Groupe 2: patients n’ayant pas développé d’érythrocytose.

L’érythrocytose est définie par un taux d’hématocrite supérieur à 51% selon les recommandations de KDIGO.

Afin de déterminer les facteurs de risque d’apparition de l’érythrocytose, nous avons comparé chez ces patients des paramètres démographiques, cliniques et biologiques à savoir l’âge, le sexe, la néphropathie initiale, la durée en hémodialyse (HD), le type de donneur (vivant ou cadavérique), la durée de la TR, le traitement immunosuppresseur, la survenue d’un rejet aigu ou chronique et l’existence d’une sténose de l’artère du greffon rénal.

Le logiciel SPSS a été utilisé pour l’analyse des données. Les variables qualitatives ont été exprimées en nombre et en pourcentage en utilisant le test de Student, et les variables quantitatives ont été exprimées en moyenne ± écart type en utilisant les testes non paramétriques. Une valeur de p <0,05 est considérée comme significative.

## Résultats

Nous avons recensé 74 TR suivis dans notre formation, 33 femmes et 41 hommes. L’âge moyen des patients était de 37±12 ans. Il s’agissait d’une allogreffe à partir d’un donneur vivant apparenté dans 65 cas (87,8%) et d’une greffe par rein de cadavre dans 9 cas (12,2%). La durée moyenne de la TR était de 37±27 mois. La néphropathie initiale était dans plus de la moitié des cas indéterminée, d’origine glomérulaire dans 21,6% des cas, secondaire à une uropathie dans 12,2% des cas, au diabète dans 5,4% des cas, à une polykystose renale (PKR) dans 1 cas, à une néphroangiosclérose dans 1 cas et à une néphrite-tubulo-interstitielle dans 1 cas.

L’érythrocytose a été notée chez 11 patients (7 hommes et 4 femmes), soit une prévalence estimée à 14,8 %. L’âge moyen des patients était de 40 ± 11 ans. Le délai d’apparition était de 9 ± 7 mois par rapport à la date de la TR. La durée de cette érythrocytose était de 6,55 ± 7 mois. La néphropathie initiale montrait une glomérulonéphrite chronique dans 3 cas, une néphropathie diabétique dans 1 cas mais reste indéterminée dans 7 cas. Un seul patient a un antécédent de tabagisme chronique sevré avant TR. Dix patients sont hypertendus, équilibrés sous monothérapie dans 2 cas, bithérapie dans 7 cas et trithérapie dans 1 cas. Trois patients ont une sténose de l’artère du greffon rénal.

Le traitement immunosuppresseur reçu consistait en la combinaison Tacrolimus- mycophénolate mofétil-corticoïde chez un patient, ciclosporinemycophénolate mofétil-corticoïde chez 4 patients et ciclosporine-azathioprine-corticoïde chez 6 patients.

Le taux moyen d’hémoglobine (Hb) était de 17 ± 0.4 g/dl (16 - 17,6 g/dl) avec un taux moyen d’hématocrite (Hte) de 52 ± 0,9 %, (51 - 54%). La créatinine sérique moyenne était de 12 ± 3 g/l avec une clairance de la créatinine (Cl cr) à 41 ± 17 ml /min/1,73m^2^ selon la formule de Cockroft et Gault (Tableau 1).

Neuf patients ont reçu un inhibiteur de l’enzyme de conversion (IEC), 4 ont bénéficié de saignées, (avec une moyenne de 3.2 ± 3 saignées). L’association des 2 méthodes thérapeutiques a été préconisée chez 2 patients. La rémission a été obtenue dans tous les cas, après une durée de 6,5 mois avec des extrêmes de 1 à 24 mois. Aucune complication thromboembolique n’a été observée. La comparaison des 2 groupes avec et sans ETR ne retrouve aucun facteur prédictif de la survenue d’ETR dans notre série (Tableau 2).

**Tableau 1:** Evolution biologique du groupe des TR ayant développé une érythrocytose

	Hb (g/dl)	Hte (%)	Urémie (g/l)	Créatininémie (mg/l)	Cl (ml/min)	cr
1 mois après TR	12 ± 2	36 ± 5	0.5 ± 0.2	15 ± 6	31 ± 15
Au moment du diagnostic de l’ETR	17 ± 0.4	52 ± 0,9	0.4 ± 0.1	12 ± 3	41 ± 17
Après traitement	13 ± 1	42 ± 5	0.4 ± 0.5	14 ± 4	31 ± 13

TR : Transplantation rénaux, ETR : Erythrocytose après transplantation rénale, Cl cr : Clearance de la créatinine. Hb : Taux d’hémoglobine, Hte : Hématocrite

**Tableau 2:** comparaison des différents paramètres chez les 2 groupes

Paramètres	Groupe 1 (n= 11)	Groupe2 (n=63)	*p*
Age	40 ± 11	36 ± 13	NS
Sexe Ratio (Homme/Femme)	7/4	34/29	NS
Durée HD	28 ± 13	30 ± 27	NS
Durée transplantation rénale	42 ± 33	36 ± 26	NS
Rejet aigu	18 %	17 %	NS
Rejet chronique	18 %	7 %	NS
Sténose de l’artère du greffon	27 %	24 %	NS
Clearance créatinine 1 mois	31 ± 15	30 ± 12	NS
Clearance créatinine actuelle	41 ± 17	39 ± 16	NS

NS: Non significatif

**Tableau 3:** le caractère thérapeutique et évolutif de notre série et de deux séries de la littérature.

	notre série n= 11 (2008)	Abdelrahman et coll n=47 (2004) [1]	Einollahi et coll n=101 (2005) [2]
Traitement	saignées + IEC	saignées + IEC	Non étudié
Rémission	100%	100%	100%
Complication	0	1 thrombophlébite	0

1 Abdelrahman M, Rafi A, Ghacha R, Qayyum T, Karkar A. Post-transplant erythrocytosis: a review of 47 renal transplant recipients. Saudi J Kidney Dis Transpl. 2004,15(4):433-9.

2 Einollahi B, Lessan-Pezeshki M, Nafar M, Pour-Reza-Gholi F, Firouzan A, Farhangi F. Erythrocytosis after renal transplantation: review of 101 cases. Transplant Proc. 2005 Sep; 37(7):3101-2.

## Discussion

L’ETR est une complication fréquente, dont la prévalence varie de 5% à 20% selon les séries [1-3]. En effet, dans notre série, la prévalence est estimée à 14.8%.

Elle survient souvent au cours de la première année de la greffe rénale [4,5]. Le délai d’apparition varie de 9 à 11 mois. Sa durée totale, depuis son apparition jusqu’à la rémission est de 6,55 ± 7 mois dans notre série, elle est courte comparativement à la série de Abdelrahman [1] où cette durée est de 37.3 ± 3 mois.

La prédominance masculine est nette [1,2,5,6], il en est de même pour notre série. Ceci est expliqué par l’effet inhibiteur des œstrogènes sur l’érythropoïèse. L’âge n’est pas retenu comme un facteur de risque dans les différentes séries [6] de même que dans la notre où l’âge moyen du groupe 1 est de 40 ± 11 ans vs 36 ± 13 ans pour le groupe 2.

La durée en HD et en TR n’a pas d’influence sur l’érythrocytose. Elle est respectivement de 28 ± 13 et de 42 ± 33 mois dans le groupe 1 vs 30 ± 27 et 36 ± 26 mois dans le groupe 2 (Tableau 2).

La néphropathie initiale peut être incriminée dans la survenue de l’érythrocytose après TR, surtout quand il s’agit d’une polykystose renale, d’une glomérulonéphrite chronique ou d’une néphro-angiosclérose [6, 7]. Dans notre série, dans le groupe 1, nous avons noté 3 cas de glomérulonéphrite chronique, et aucun cas de polykystose renale ni de néphro-angiosclérose.

Le traitement immunosuppresseur, notamment les corticoïdes et la ciclosporine, est impliqué dans le survenue de l’ETR. Ils semblent induire une importante capacité de multiplication des cellules souches hématopoïétiques [7].

La ciclosporine pourrait stimuler la production de l’EPO de façon directe par vasoconstriction des artères afférentes, et de façon indirecte par l’inhibition de la synthèse rénale des prostaglandines. Dans notre série, tous nos patients sont sous corticoïdes et ciclosporine. Un patient du groupe 1 a été switché au tacrolimus 15 jours après la greffe rénale, une érythrocytose est apparue 18 mois après, un traitement à base d’IEC et d’une saignée a été instauré, la rémission a été obtenue 3 mois plus tard.

Le rejet aigu ou chronique induit une hypoxie locale et donc une hypersécrétion d’EPO [4,7]. Dans notre série le rejet n’était pas retrouvé comme facteur de risque de développement d’une érythrocytose.

La sténose de l’artère du greffon rénal est un facteur prédisposant à l’ETR par le biais d’une ischémie rénale [8]. La sténose artérielle est notée dans 27% des patients du groupe 1 vs 24% des patients du groupe 2, sans aucune différence significative.

Le tabagisme est rapporté comme facteur prédisposant à l’érythrocytose [8]. Dans notre série un seul patient était tabagique mais sevré avant la greffe rénale. L’érythrocytose ne semble pas influencer la fonction du greffon rénal. En effet la comparaison des 2 groupes selon la présence ou non de l’érythrocytose n’a pas montré de différence significative de clairance de la créatinine dans la série de Abdelrahman [1].

De même, dans notre série, nous n’avons pas noté de dégradation de la fonction rénale après apparition de l’érythrocytose.

Notre étude reste limitée par le nombre réduit de malade ayant développé une ETR.

Les saignées itératives permettent de contrôler plus rapidement l’ETR et diminuent le risque de complication vasculaire. Le volume sanguin à prélever par saignée varie de 100 à 500 ml, répété en moyenne trois fois en 2 semaines jusqu’à atteindre un taux d’hématocrite inférieur à 45%. Quatre patients de notre série ont nécessité des saignées.

Le traitement médical de l’ETR repose sur les IEC et les antagonistes des récepteurs de l’angiotensine 2 (ARA 2). Le mécanisme d’action de ces molécules n’est pas bien connu, ils agiraient en inhibant la synthèse de l’érythropoïétine et auraient l’avantage de lutter contre l’ischémie et l’hypoxie médullaire par vasodilatation artérielle [8-13].

Ces médicaments sont contre-indiqués en cas de sténose de l’artère du greffon rénal. Dans notre étude, l’administration d’un IEC a permis de diminuer les taux d’Hb et d’Ht, au bout de 6.5±7 mois.

La théophylline peut être utilisée dans le traitement de l’érythrocytose, son utilisation reste limitée car elle potentialise l’action de la ciclosporine [14].

La néphrectomie bilatérale a également été proposée dans le traitement de la PG [15] puisqu’on incrimine les reins natifs dans l’hyperproduction de l’EPO [8,16]. Cette alternative est abandonnée au bénéfice de nouvelles molécules qui sont les IEC et les ARA 2.

La rémission a été obtenue chez tous nos patients sans aucune complication thromboembolique. Ceci pourrait s’expliquer par le fait que le taux d’hématocrite initial n’est pas très élevé. Cette rémission a été obtenue dans 100 % des cas dans la série d’Einollahi [2], de même que dans la série de Abdelrahman [1] mais avec 1 cas de thrombose veineuse profonde du membre inférieur dans cette dernière (Tableau 3).

## Conclusion

L’ETR est une complication fréquente. Elle survient au cours de la première année de la greffe rénale. La gravité est reflétée par la survenue de complication thrombo-embolique qui reste rare. Le pronostic du greffon est souvent bon. Les IEC ont nettement amélioré la qualité de la prise en charge notamment en cas d’érythrocytose non sévère.

## Conflits d’intérêts

Nous n’avons aucun conflit d’intérêts.

## Contribution des auteurs

Tous les auteurs ont participé á la prise en charge des patients et a la rédaction du manuscrit.
